# Sex Disparities in Outcomes of Cardiogenic Shock Complicating Non–ST-Segment-Elevation Myocardial Infarction

**DOI:** 10.1016/j.jscai.2026.105383

**Published:** 2026-05-20

**Authors:** Hritvik Jain, Nandan Patel, Jyoti Jain, Siddharth P. Agrawal, Allison Dupont, Alexander G. Truesdell, Srihari S. Naidu, J. Dawn Abbott, Saraschandra Vallabhajosyula

**Affiliations:** a Department of Medicine, All India Institute of Medical Sciences, Jodhpur, India; b Department of Internal Medicine, Allegheny General Hospital, Pittsburgh, Pennsylvania; c Department of Internal Medicine, New York Medical College/Landmark Medical Center, Woonsocket, Rhode Island; d Northside Hospital Cardiovascular Institute, Atlanta, Georgia; e Virginia Heart/Inova Schar Heart and Vascular, Falls Church, Virginia; f Division of Cardiovascular Medicine, Department of Medicine, Westchester Medical Center and New York Medical College, Valhalla, New York; g Division of Cardiology, Department of Medicine, Warren Alpert Medical School of Brown University, Providence, Rhode Island; h Brown University Health Cardiovascular Institute, Providence, Rhode Island

**Keywords:** non–ST-segment-elevation myocardial infarction, cardiogenic shock, sex disparities, acute coronary syndromes

Non–ST-segment-elevation myocardial infarction complicated by cardiogenic shock (NSTEMI-CS) continues to be associated with high morbidity and mortality.^1^ While sex differences in presentation, management, and outcomes have been well described in acute myocardial infarction (AMI) and cardiogenic shock (CS) independently, data specifically examining sex disparities within NSTEMI-CS remain limited.^2^ Women with NSTEMI often present at an older age with comorbidities, differences in coronary and vascular anatomy, and risk profiles, further contributing to outcome disparities in this population.^3^^,^^4^

This analysis was conducted using the United States Collaborative Network of the TriNetX database (Cambridge, MA), which includes data from 70+ health care organizations. Ethical approval was exempt from the Western Institutional Review Board, as the TriNetX platform is compliant with the Health Insurance Portability and Accountability Act Privacy Rule (Section §164.514). Patients ≥18 years with CS (International Classification of Diseases, Tenth Edition [ICD-10] code R57.0) occurring within −2 to +7 days after NSTEMI (ICD-10 I21.4) were identified between January 01, 2010, and December 22, 2025, with the date of diagnosis of CS as the ‘index event’ in both the cohorts.^5^ The primary outcome was all-cause mortality, while the secondary outcomes included recurrent AMI, heart failure (HF) exacerbation, ischemic stroke, palliative care consultation, do-not-intubate (DNI) or do-not-resuscitate (DNR) status, mechanical circulatory support (MCS) utilization, major bleeding, major vascular complications, and all-cause readmission at 30 days and 1 year. All-cause mortality was evaluated across prespecified subgroups, including age (<65 years and ≥65 years), race (White/non-White), MCS utilization, and cardiac arrest. Sensitivity analyses were performed for study era (2010-2017 vs 2018-2025) for all-cause mortality, and by baseline HF and percutaneous coronary intervention (PCI) for MCS utilization.

Continuous and categorical variables are presented as mean ± SD and n (%) and were compared using the independent-sample *t* test and χ^2^ test, respectively. We utilized 1:1 propensity-score matching (PSM) across demographics, comorbidities, medications, and laboratory parameters, using logistic regression via the “greedy nearest-neighbor matching” method, with a caliper of 0.1 pooled standard deviations of the linear propensity scores. Covariates were deemed balanced if standardized mean differences were <0.1. Cox proportional hazard models estimated hazard ratios (HR) with 95% CI. Effect estimates were also reported in risk differences (RD) with 95% CI. Statistical analyses were conducted within the R computing software.

During the time period from January 01, 2010, to December 22, 2025, of 58,524 NSTEMI-CS patients, 21,872 (31.3%) were women. Before PSM, women were, on average, older, of non-white race, and lower PCI utilization (Supplemental Table S1). Following PSM, both cohorts had 19,957 patients—mean age 69.2 ± 12.8 years and White race 69.9%.

In the matched cohort analysis at 30 days, compared to men, women had higher all-cause mortality (HR 1.06; 95% CI, 1.03-1.10; RD 1.3% [0.3, 2.2]; *P* < 0.001) and major bleeding (HR 1.04; 95% CI, 1.01-1.09; RD 0.8% [0.2, 1.4]; *P* = 0.019); however, they had lower rates of HF exacerbation (HR 0.96; 95% CI, 0.93-0.98; RD −2.4% [−3.3, −1.5]; *P* < 0.001) and MCS utilization (HR 0.86; 95% CI, 0.82-0.91; RD −2.1% [−2.8, −1.4]; *P* < 0.001). No difference in other secondary outcomes was noted (Figure 1A).

**Figure 1:**
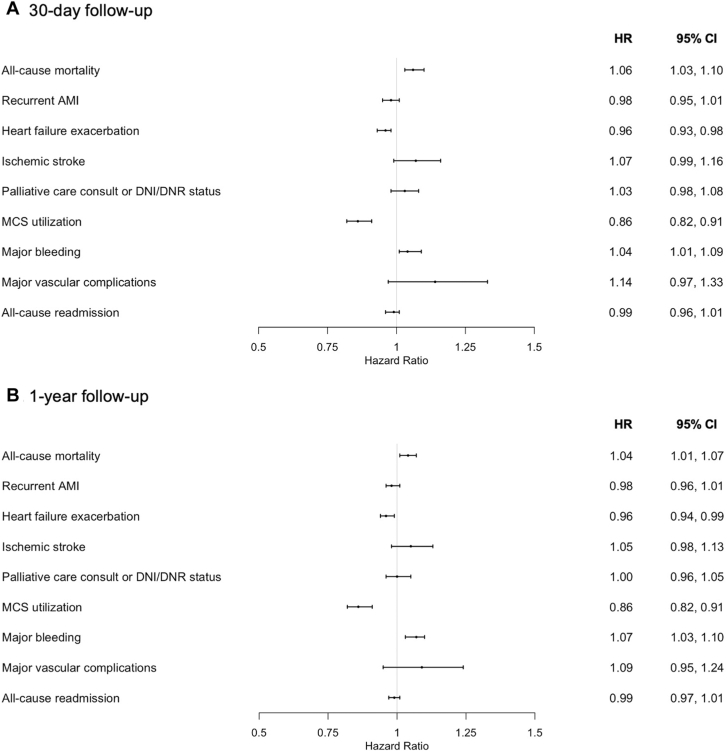

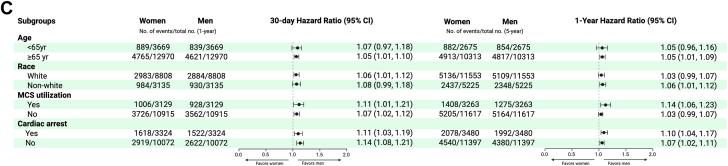
**NSTEMI-CS outcomes between women and men at 30 days.** (**A**) 1year, (**B**) follow-up, (**C**) prespecified subgroup analysis for all-cause mortality. AMI, acute myocardial infarction; DNI, do-not-intubate; DNR, do-not-resuscitate; HR, hazard ratio; MCS, mechanical circulatory support.

In stratified analyses, women had lower MCS utilization in those not undergoing PCI (HR 0.80; 95% CI, 0.75-0.86; *P* < 0.001) and with baseline HF (HR 0.86; 95% CI, 0.81-0.91; *P* < 0.001). The observed differences in mortality between sexes were consistent across study eras: 2010 to 2017 (HR 1.13; 95% CI, 1.05-1.22; *P* < 0.001) vs 2018 to 2025 (HR 1.06; 95% CI, 1.02-1.11; *P* = 0.002). At 1-year follow-up, similar and consistent trends were noted (Figure 1B). In subgroup analysis for all-cause mortality, similar trends were noted, except in age <65 years, White race, and those without MCS utilization (Figure 1C).

In NSTEMI-CS, women had higher rates of short- and long-term mortality, bleeding risk, lower rates of MCS use, and recurrent HF. The rates of recurrent AMI were comparable.

Clinically, women with NSTEMI-CS often present later in the disease course, with more advanced hemodynamic compromise, greater frailty, and reduced physiologic reserve, all of which narrow the margin for hemodynamic compensation and tolerance to prolonged low-output states. In addition, women are more likely to manifest shock with less overt hypotension, potentially leading to under recognition and delayed intervention.^1^ Although statistically significant, the absolute difference in mortality between sexes was modest and must be interpreted cautiously. Lower utilization of MCS in women was observed in this analysis, which is consistent with prior studies; however, whether this difference contributes to outcome disparity cannot be determined from the available data.^2^ Reduced MCS use in women likely reflects conservative clinical decision making, driven by concerns regarding age, bleeding risk, body size, vascular access, or perceived procedural futility. Alternative explanations may also contribute to treatment differences, including variations in ventricular geometry, smaller left ventricular cavity size, differing shock phenotype, or contraindications to advanced MCS. Notably, the difference in MCS utilization was confined to patients who did not undergo PCI, whereas MCS use was similar between sexes once an invasive strategy was pursued. However, the similar rates of vascular complications observed between sexes suggest that procedural risk alone does not justify this disparity.^3^ Women also experienced a modest but significant increase in major bleeding, consistent with prior AMI literature. This likely reflects a combination of older age, renal dysfunction, and antithrombotic exposure rather than procedural complications, particularly given the similar rates of vascular injury.

This study has multiple limitations. Due to its retrospective observational design and administrative coding, causal inference cannot be interpreted. The TriNetX dataset lacks granular clinical details on angiographic findings and revascularization strategy, timing/escalation of vasoactive therapies, invasive hemodynamic parameters, shock trajectory, and standardized measures of illness severity, all of which may influence treatment decisions. Given the TriNetX interface limitations, formal competing risk regression could not be performed; thus, nonfatal outcomes were analyzed with death, treated as a censoring event. Additionally, the predefined window used to identify CS relative to NSTEMI may introduce clinical heterogeneity, as cases in which shock precedes NSTEMI may reflect alternative etiologies.

In conclusion, women with NSTEMI-CS demonstrated higher mortality, bleeding, and lower MCS use compared to men. Collectively, these findings support the need for sex-agnostic protocols prioritizing earlier recognition, objective hemodynamic assessment, and standardized escalation.

## Declaration of competing interest

Alexander Truesdell reports consulting/speaking fees/honoraria from Abiomed, Chiesi, Getinge, and Zoll. J. Dawn Abbott receives research funding from Boston Scientific, Shockwave Medical, Novo Nordisk, and MedAlliance, and serves as a consultant for Abbott, Medtronic, Boston Scientific, and Recor Medical. Hritvik Jain, Nandan Patel, Jyoti Jain, Siddharth Agrawal, Allison Dupont, Srihari Naidu, and Saraschandra Vallabhajosyula report no financial interests.

## Footnotes

To access the supplementary material accompanying this article, visit the online version of the *Journal of the Society for Cardiovascular Angiography & Interventions* at 10.1016/j.jscai.2026.105383.
