# Supplementary material for: Sex Disparities in Outcomes of Cardiogenic Shock Complicating Non–ST-Segment-Elevation Myocardial Infarction
Source: J Soc Cardiovasc Angiogr Interv. 2026 May 20;5(7):105383. doi: 10.1016/j.jscai.2026.105383 (PMC13400107; doi:10.1016/j.jscai.2026.105383)
Supplement: Supplementary Table S1 [file mmc1.docx]

**SUPPLEMENTARY MATERIAL**

**Sex Disparities in Outcomes of Cardiogenic Shock Complicating Non-ST-Segment-Elevation Myocardial Infarction**

Hritvik Jain, MBBS; Nandan Patel, MBBS; Jyoti Jain, MD; Siddharth P Agrawal, MD; Allison Dupont, MD; Alexander G Truesdell, MD; Srihari S Naidu, MD; J Dawn Abbott, MD; Saraschandra Vallabhajosyula, MD MSc

**Supplementary Table S1. Baseline characteristics of the study cohort before and after propensity score matching**

| **Variables** | | **Before propensity-score matching** | | | | **After propensity-score matching** | | | |
| --- | --- | --- | --- | --- | --- | --- | --- | --- | --- |
|  |  | **Women**  **(n = 21,872)** | **Men**  **(n = 36,652)** | **P value** | **Std. difference** | **Women**  **(n = 19,957)** | **Men**  **(n = 19,957)** | **P value** | **Std. difference** |
| Current age | | 74.6 ± 12.7 | 72.7 ± 12.5 | <0.01 | 0.150 | 74.2 ± 12.8 | 74.3 ± 12.1 | 0.63 | 0.005 |
| Age at index event | | 69.7 ± 13.0 | 67.7 ± 12.7 | <0.01 | 0.155 | 69.2 ± 13.1 | 69.3 ± 12.4 | 0.72 | 0.004 |
| Race | White | 14,962 (68.4) | 26,281 (71.7) | <0.01 | 0.072 | 13,921 (69.8) | 13,963 (70) | 0.65 | 0.005 |
|  | African American | 4,045 (18.5) | 4,887 (13.3) | <0.01 | 0.141 | 3,313 (16.6) | 3,306 (16.6) | 0.93 | 0.001 |
|  | Asian | 917 (4.2) | 1,829 (5) | <0.01 | 0.038 | 879 (4.4) | 901 (4.5) | 0.59 | 0.005 |
|  | Others | 939 (4.3) | 1,825 (5) | <0.01 | 0.033 | 889 (4.5) | 865 (4.3) | 0.56 | 0.006 |
| Comorbidities | Hypertension | 14,661 (67) | 22,893 (62.5) | <0.01 | 0.096 | 12,888 (64.6) | 12,755 (63.9) | 0.17 | 0.014 |
|  | Heart failure | 9,356 (42.8) | 15,425 (42.1) | 0.10 | 0.014 | 8,296 (41.6) | 8,159 (40.9) | 0.16 | 0.014 |
|  | Atrial fibrillation | 5,132 (23.5) | 9,128 (24.9) | <0.01 | 0.034 | 4,639 (23.2) | 4,574 (22.9) | 0.44 | 0.008 |
|  | Cardiomyopathy | 3,534 (16.2) | 6,962 (19) | <0.01 | 0.075 | 3,246 (16.3) | 3,211 (16.1) | 0.63 | 0.005 |
|  | Aortic valve disorders | 3,474 (15.9) | 5,531 (15.1) | 0.01 | 0.022 | 3,038 (15.2) | 2,937 (14.7) | 0.16 | 0.014 |
|  | Mitral valve disorders | 4,464 (20.4) | 6,028 (16.4) | <0.01 | 0.102 | 3,726 (18.7) | 3,600 (18) | 0.10 | 0.016 |
|  | Cardiac arrest | 1,057 (4.8) | 2,069 (5.6) | <0.01 | 0.036 | 976 (4.9) | 999 (5) | 0.60 | 0.005 |
|  | Ischemic heart diseases | 12,012 (54.9) | 20,924 (57.1) | <0.01 | 0.044 | 10,873 (54.5) | 10,754 (53.9) | 0.23 | 0.012 |
|  | Peripheral vascular diseases | 3,800 (17.4) | 6,155 (16.8) | 0.07 | 0.015 | 3,370 (16.9) | 3,352 (16.8) | 0.81 | 0.002 |
|  | Stroke | 2,602 (11.9) | 3,490 (9.5) | <0.01 | 0.077 | 2,165 (10.8) | 2,159 (10.8) | 0.92 | 0.001 |
|  | Diabetes mellitus | 9,317 (42.6) | 14,385 (39.2) | <0.01 | 0.068 | 8,229 (41.2) | 8,128 (40.7) | 0.30 | 0.010 |
|  | Dyslipidemia | 12,468 (57) | 19,675 (53.7) | <0.01 | 0.067 | 10,984 (55) | 10,855 (54.4) | 0.20 | 0.013 |
|  | Osteoarthritis | 6,375 (29.1) | 7,369 (20.1) | <0.01 | 0.211 | 5,048 (25.3) | 5,002 (25.1) | 0.60 | 0.005 |
|  | Chronic kidney disease | 7,076 (32.4) | 11,498 (31.4) | 0.01 | 0.021 | 6,267 (31.4) | 6,168 (30.9) | 0.29 | 0.011 |
|  | Lower respiratory diseases | 6,776 (31) | 9,003 (24.6) | <0.01 | 0.144 | 5,637 (28.2) | 5,555 (27.8) | 0.36 | 0.009 |
|  | Liver diseases | 2,980 (13.6) | 5,029 (13.7) | 0.74 | 0.003 | 2,600 (13) | 2,612 (13.1) | 0.86 | 0.002 |
|  | Sleep apnea | 2,869 (13.1) | 5,636 (15.4) | <0.01 | 0.065 | 2,626 (13.2) | 2,583 (12.9) | 0.52 | 0.006 |
|  | Smoking | 4,186 (19.1) | 7,835 (21.4) | <0.01 | 0.056 | 3,827 (19.2) | 3,736 (18.7) | 0.25 | 0.012 |
|  | Alcohol use disorders | 699 (3.2) | 2,872 (7.8) | <0.01 | 0.204 | 699 (3.5) | 692 (3.5) | 0.85 | 0.002 |
|  | Neoplasms | 6,322 (28.9) | 9,460 (25.8) | <0.01 | 0.069 | 5,474 (27.4) | 5,482 (27.5) | 0.93 | 0.001 |
| Prior procedures | PPM/ICD | 928 (4.2) | 2,101 (5.7) | <0.01 | 0.068 | 869 (4.4) | 866 (4.3) | 0.94 | 0.001 |
|  | CABG | 430 (2) | 895 (2.4) | <0.01 | 0.032 | 405 (2) | 402 (2) | 0.92 | 0.001 |
|  | Dialysis | 1,293 (5.9) | 2,060 (5.6) | 0.14 | 0.012 | 1,156 (5.8) | 1,152 (5.8) | 0.93 | 0.001 |
|  | PCI | 1,867 (8.5) | 3,586 (9.8) | <0.01 | 0.043 | 1,736 (8.7) | 1,684 (8.4) | 0.35 | 0.009 |
| Medications | Beta blockers | 12,541 (57.3) | 19,808 (54) | <0.01 | 0.066 | 10,994 (55.1) | 10,884 (54.5) | 0.27 | 0.011 |
|  | Antilipemic agents | 12,196 (55.8) | 19,870 (54.2) | <0.01 | 0.031 | 10,842 (54.3) | 10,714 (53.7) | 0.20 | 0.013 |
|  | Antiarrhythmics | 11,875 (54.3) | 18,821 (51.4) | <0.01 | 0.059 | 10,418 (52.2) | 10,329 (51.8) | 0.37 | 0.009 |
|  | Diuretics | 11,755 (53.7) | 17,665 (48.2) | <0.01 | 0.111 | 10,155 (50.9) | 10,084 (50.5) | 0.48 | 0.007 |
|  | MRAs | 2,874 (13.1) | 4,963 (13.5) | 0.17 | 0.012 | 2,542 (12.7) | 2,491 (12.5) | 0.44 | 0.008 |
|  | ACE/ARBs/ARNI | 12,954 (59.2) | 20,726 (56.6) | 0.06 | 0.016 | 11,340 (56.8) | 11,182 (56) | 0.39 | 0.009 |
|  | Calcium channel blockers | 8,801 (40.2) | 13,105 (35.8) | <0.01 | 0.092 | 7,541 (37.8) | 7,520 (37.7) | 0.83 | 0.002 |
|  | Antianginals | 7,849 (35.9) | 13,497 (36.8) | 0.02 | 0.020 | 7,057 (35.4) | 6,947 (34.8) | 0.25 | 0.012 |
|  | Bronchodilators | 11,804 (54) | 17,516 (47.8) | <0.01 | 0.124 | 10,199 (51.1) | 10,100 (50.6) | 0.32 | 0.010 |
|  | Insulin | 8,944 (40.9) | 13,735 (37.5) | <0.01 | 0.070 | 7,834 (39.3) | 7,766 (38.9) | 0.49 | 0.007 |
|  | SGLT2i | 1,706 (7.8) | 3,456 (9.4) | <0.01 | 0.036 | 1,567 (7.9) | 1,536 (7.7) | 0.89 | 0.001 |
|  | Anticoagulants | 12,925 (59.1) | 20,409 (55.7) | <0.01 | 0.069 | 11,326 (56.8) | 11,217 (56.2) | 0.27 | 0.011 |
|  | Enoxaparin | 6,872 (31.4) | 9,599 (26.2) | <0.01 | 0.116 | 5,789 (29) | 5,751 (28.8) | 0.68 | 0.004 |
|  | Apixaban | 2,626 (12) | 4,355 (11.9) | 0.65 | 0.004 | 2,318 (11.6) | 2,250 (11.3) | 0.29 | 0.011 |
|  | Warfarin | 2,073 (9.5) | 3,466 (9.5) | 0.93 | 0.001 | 1,820 (9.1) | 1,791 (9) | 0.61 | 0.005 |
|  | Rivaroxaban | 804 (3.7) | 1,476 (4) | 0.03 | 0.018 | 728 (3.6) | 692 (3.5) | 0.33 | 0.010 |
|  | Platelet aggregation inhibitors | 12,017 (54.9) | 19,765 (53.9) | 0.02 | 0.020 | 10,681 (53.5) | 10,550 (52.9) | 0.19 | 0.013 |
|  | Aspirin | 11,560 (52.9) | 19,047 (52) | 0.04 | 0.018 | 10,285 (51.5) | 10,161 (50.9) | 0.21 | 0.012 |
|  | Clopidogrel | 5,574 (25.5) | 9,647 (26.3) | 0.03 | 0.019 | 5,053 (25.3) | 4,984 (25) | 0.43 | 0.008 |
|  | Ticagrelor | 848 (3.9) | 1,552 (4.2) | 0.04 | 0.018 | 770 (3.9) | 753 (3.8) | 0.66 | 0.004 |
|  | Prasugrel | 306 (1.4) | 682 (1.9) | <0.01 | 0.036 | 293 (1.5) | 312 (1.6) | 0.44 | 0.008 |
|  | Glucocorticoids | 11,397 (52.1) | 16,273 (44.4) | <0.01 | 0.155 | 9,771 (49) | 9,664 (48.4) | 0.28 | 0.011 |
| In-hospital management | Heparin | 10,807 (49.4) | 17,389 (47.4) | <0.01 | 0.039 | 9,496 (47.6) | 9,403 (47.1) | 0.35 | 0.009 |
|  | Bivalirudin | 960 (4.4) | 1,725 (4.7) | 0.08 | 0.015 | 873 (4.4) | 871 (4.4) | 0.96 | <0.001 |
|  | Eptifibatide | 388 (1.8) | 785 (2.1) | <0.01 | 0.027 | 368 (1.8) | 372 (1.9) | 0.88 | 0.001 |
|  | Tirofiban | 141 (0.6) | 315 (0.9) | <0.01 | 0.025 | 135 (0.7) | 131 (0.7) | 0.81 | 0.002 |
|  | Fondaparinux | 130 (0.6) | 142 (0.4) | <0.01 | 0.030 | 100 (0.5) | 103 (0.5) | 0.83 | 0.002 |
|  | Cangrelor | 94 (0.4) | 206 (0.6) | 0.03 | 0.019 | 86 (0.4) | 98 (0.5) | 0.38 | 0.009 |
|  | Epinephrine | 5,302 (24.2) | 8,533 (23.3) | 0.01 | 0.023 | 4,635 (23.2) | 4,639 (23.2) | 0.96 | <0.001 |
|  | Phenylephrine | 4,953 (22.6) | 7,918 (21.6) | <0.01 | 0.025 | 4,337 (21.7) | 4,312 (21.6) | 0.76 | 0.003 |
|  | Norepinephrine | 2,954 (13.5) | 5,083 (13.9) | 0.22 | 0.011 | 2,681 (13.4) | 2,679 (13.4) | 0.98 | <0.001 |
|  | Dobutamine | 1,186 (5.4) | 2,225 (6.1) | <0.01 | 0.028 | 1,084 (5.4) | 1,072 (5.4) | 0.79 | 0.003 |
|  | Dopamine | 889 (4.1) | 1,447 (3.9) | 0.49 | 0.006 | 799 (4) | 804 (4) | 0.90 | 0.001 |
|  | Sodium, mmol/L | 137.7 ± 4.2 | 137.4 ± 4.1 | <0.01 | 0.062 | 137.6 ± 4.2 | 137.5 ± 4.0 | 0.07 | 0.021 |
|  | Potassium, mmol/L | 4.2 ± 0.6 | 4.2 ± 0.6 | <0.01 | 0.109 | 4.2 ± 0.6 | 4.2 ± 0.6 | <0.01 | 0.113 |
|  | Chloride, mmol/L | 101.6 ± 5.6 | 101.6 ± 5.5 | 0.14 | 0.015 | 101.6 ± 5.7 | 101.7 ± 5.5 | 0.60 | 0.006 |
|  | Bicarbonate, mmol/L | 24.8 ± 4.8 | 24.6 ± 4.5 | <0.01 | 0.036 | 24.7 ± 4.7 | 24.7 ± 4.6 | 0.87 | 0.002 |
|  | Urea nitrogen, mg/dL | 27.0 ± 18.7 | 29.4 ± 20.4 | <0.01 | 0.122 | 27.0 ± 18.7 | 29.4 ± 20.4 | <0.01 | 0.123 |
|  | Creatinine, mg/dL | 1.7 ± 2.3 | 2.0 ± 3.5 | <0.01 | 0.112 | 1.7 ± 2.4 | 2.0 ± 3.3 | <0.01 | 0.109 |
|  | Glucose, mg/dL | 134.4 ± 66.9 | 133.9 ± 63.6 | 0.44 | 0.008 | 135.0 ± 67.8 | 133.7 ± 62.5 | 0.08 | 0.021 |
|  | Calcium, mg/dL | 9.0 ± 0.8 | 8.9 ± 0.7 | <0.01 | 0.1 | 9.0 ± 0.8 | 8.9 ± 0.7 | <0.01 | 0.091 |
|  | Platelets, K/uL | 238.9 ± 100.0 | 216.5 ± 91.9 | <0.01 | 0.234 | 238.7 ± 99.8 | 217.6 ± 92.0 | <0.01 | 0.22 |
|  | Alanine aminotransferase, IU/L | 53.3 ± 249.1 | 62.1 ± 260.4 | <0.01 | 0.034 | 53.0 ± 240.2 | 58.1 ± 231.9 | 0.08 | 0.022 |
|  | Aspartate aminotransferase, IU/L | 77.6 ± 476.5 | 75.7 ± 373.8 | 0.66 | 0.004 | 77.2 ± 448.8 | 69.9 ± 338.7 | 0.14 | 0.018 |
|  | Alkaline phosphatase, IU/L | 102.3 ± 74.8 | 99.2 ± 76.0 | <0.01 | 0.041 | 102.4 ± 75.5 | 99.9 ± 82.1 | 0.01 | 0.032 |
|  | Lactate dehydrogenase, IU/L | 403.6 ± 1174.5 | 392.3 ± 699.7 | 0.57 | 0.012 | 388.5 ± 934.7 | 381.8 ± 700.0 | 0.76 | 0.008 |
|  | Total bilirubin, mg/dL | 0.7 ± 0.9 | 0.8 ± 1.1 | <0.01 | 0.158 | 0.7 ± 0.9 | 0.8 ± 1.1 | <0.01 | 0.133 |
|  | Direct bilirubin, mg/dL | 0.3 ± 0.7 | 0.4 ± 1.0 | <0.01 | 0.106 | 0.3 ± 0.7 | 0.4 ± 0.9 | <0.01 | 0.085 |
|  | Albumin, g/dL | 3.5 ± 0.7 | 3.6 ± 0.7 | <0.01 | 0.063 | 3.5 ± 0.7 | 3.5 ± 0.7 | 0.03 | 0.028 |
|  | Cholesterol, mg/dL | 165.4 ± 56.1 | 148.2 ± 49.7 | <0.01 | 0.324 | 165.6 ± 56.8 | 148.1 ± 48.6 | <0.01 | 0.33 |
|  | Low-density lipoprotein, mg/dL | 90.2 ± 44.6 | 82.2 ± 39.7 | <0.01 | 0.189 | 90.5 ± 45.2 | 82.4 ± 39.2 | <0.01 | 0.192 |
|  | High-density lipoprotein, mg/dL | 45.2 ± 20.5 | 37.8 ± 16.7 | <0.01 | 0.391 | 44.8 ± 20.1 | 38.2 ± 16.8 | <0.01 | 0.355 |
|  | Triglyceride, mg/dL | 143.2 ± 135.2 | 138.3 ± 118.8 | <0.01 | 0.038 | 145.4 ± 141.3 | 134.5 ± 113.6 | <0.01 | 0.085 |
|  | Troponin I, ng/mL | 2.8 ± 14.4 | 3.6 ± 16.9 | 0.01 | 0.047 | 3.0 ± 15.2 | 3.1 ± 15.5 | 0.80 | 0.005 |
|  | NT-proBNP, pg/mL | 9120.9 ± 13639.8 | 8414.6 ± 13130.0 | 0.01 | 0.053 | 9394.1 ± 13878.8 | 8492.7 ± 13003.6 | <0.01 | 0.067 |
|  | Hemoglobin A1c, % | 6.8 ± 1.9 | 6.8 ± 1.8 | 0.30 | 0.013 | 6.8 ± 1.9 | 6.8 ± 1.8 | 0.21 | 0.018 |
|  | Lactate, mmol/L | 1.9 ± 1.8 | 1.9 ± 1.8 | 0.93 | 0.001 | 1.9 ± 1.8 | 1.9 ± 1.7 | 0.46 | 0.013 |
|  | Respiratory rate, /min | 17.6 ± 3.6 | 17.7 ± 3.7 | 0.03 | 0.027 | 17.6 ± 3.6 | 17.7 ± 3.6 | 0.08 | 0.025 |
|  | Heart rate, /min | 80.7 ± 17.4 | 79.5 ± 17.8 | <0.01 | 0.068 | 80.8 ± 17.5 | 79.2 ± 17.5 | <0.01 | 0.088 |
|  | Oxygen saturation, % | 83.7 ± 23.4 | 84.4 ± 22.5 | 0.02 | 0.032 | 83.6 ± 23.4 | 84.3 ± 22.9 | 0.07 | 0.029 |
|  | Systolic pressure, mmHg | 124.4 ± 24.6 | 122.7 ± 23.7 | <0.01 | 0.069 | 124.1 ± 24.6 | 123.6 ± 23.6 | 0.09 | 0.021 |
|  | Diastolic pressure, mmHg | 68.2 ± 14.5 | 70.1 ± 14.5 | <0.01 | 0.13 | 68.2 ± 14.6 | 69.8 ± 14.3 | <0.01 | 0.11 |
|  | Body mass index, kg/m^2^ | 29.0 ± 7.8 | 28.5 ± 6.5 | <0.01 | 0.062 | 29.0 ± 7.8 | 28.2 ± 6.4 | <0.01 | 0.109 |
|  | Left ventricular ejection fraction (%) | 49.2 ± 16.9 | 43.4 ± 16.9 | <0.01 | 0.345 | 48.7 ± 17.0 | 44.7 ± 16.7 | <0.01 | 0.235 |

**Legend**: Data are presented as mean ± standard deviation or frequency (percentage).

**Abbreviations**: ACE: angiotensin-converting enzyme; CABG: coronary artery bypass grafting; ICD: implantable cardioverter defibrillator; INR: international normalized ratio; NT-proBNP: N-terminal pro-B-type natriuretic peptide; PCI: percutaneous coronary intervention; PPM: permanent pacemaker.
